# Development of a Japanese version of the Awe Experience Scale (AWE-S): A structural topic modeling approach

**DOI:** 10.12688/f1000research.134275.2

**Published:** 2023-10-13

**Authors:** Ryota Takano, Akiko Matsuo, Kazuaki Kawano

**Affiliations:** 1 Kojimachi Business Center Building, Japan Society for the Promotion of Science, Tokyo, 102-0083, Japan; 2 Department of Social Psychology, Graduate School of Humanities and Sociology, The University of Tokyo, Bunkyo-ku, Tokyo, 113-0033, Japan; 3 Research Center for Advanced Science and Technology, The University of Tokyo, Meguro-Ku, Tokyo, 153-8904, Japan; 4 Department of Psychology, Tokai Gakuen University, Nagoya, Aichi, 468-8514, Japan

**Keywords:** awe, emotion, scale, text-mining, narrative, topic modeling, culture, questionnaire

## Abstract

Background: Awe, a complex emotion, arises in response to perceptually and conceptually vast stimuli that transcend one’s current frames of reference, which is associated with subjective psychological phenomena, such as a sense of self and consciousness. This study aimed to develop a Japanese version of the Awe Experience Scale (AWE-S), a widely used questionnaire that robustly measured the state of awe, and simultaneously investigated how the multiple facets of awe related to the narrative representations of awe experiences. Methods: The Japanese AWE-S was created via back-translation and its factor structure and validity was investigated through an online survey in Japan. Results: The results revealed that the Japanese AWE-S consisted of the same six factors as the original (i.e., time, self-loss, connectedness, vastness, physiological, and accommodation) and had sufficient internal consistency, test-retest reliability, construct validity, and also Japan-specific characteristics. The structured topic modeling generated seven potential topics of the descriptions of awe experiences, which were differently associated with each factor of the Japanese AWE-S. Conclusions: Our findings contribute to a deeper understanding of awe and reveal the constructs of awe in Japan through cross-cultural comparisons. Furthermore, this study provides conceptual and methodological implications regarding studies on awe.

## Introduction

Awe is an emotional response to perceptually and conceptually vast stimuli that transcend one’s current frames of reference ( Keltner & Haidt, 2003). It has been studied in various fields, such as psychology, economics, neuroscience, immunology, and public health ( Guan *et al.*, 2018; Ibanez *et al.*, 2017; Monroy & Keltner, 2022; Piff *et al.*, 2015; Sawada & Nomura, 2020; Stellar *et al.*, 2015; Sun *et al.*, 2021; Takano & Nomura, 2022b; van Elk *et al.*, 2019). Previous studies have shown that awe is associated with subjective psychological phenomena, such as a sense of self and consciousness, which highlights the need for a standardized questionnaire to assess the various dimensions of awe experiences ( Bai *et al.*, 2017; Rudd *et al.*, 2012; Takano & Nomura, 2021). The Awe Experience Scale (AWE-S), a widely used questionnaire that robustly measures the state of awe ( Yaden *et al.*, 2019), has been translated into several languages ( Landrum *et al.*, 2022; Ling-xiao *et al.*, 2022; Rodrigues *et al.*, 2022; van Houwelingen-Snippe *et al.*, 2020). However, how the multiple facets of awe measured by the AWE-S relate to the representations of awe experiences in different cultural contexts remains unclear. Simultaneously, there is no reliable and valid standardized Japanese version of the AWE-S. Therefore, it is important to develop a standardized Japanese AWE-S and investigate its narrative dimensions not only for a deeper understanding of the complex emotion of awe and revealing the construct of awe in Japan through cross-cultural comparisons, but also for investigating the psychological mechanisms underlying the various effects of awe on mental health and social behavior ( Bai *et al.*, 2021; Liu *et al.*, 2023; Piff *et al.*, 2015; Stellar *et al.*, 2018; Sturm *et al.*, 2022; Takano & Nomura, 2021).

The AWE-S questionnaire comprises six factors: time, self-loss, connectedness, vastness, physiological, and accommodation ( Yaden *et al.*, 2019). Time captures the perception of time expansion in awe experiences, which may indicate a shift in mental state towards a further mindful, moment-to-moment awareness, such as *here and now* ( Gregory *et al.*, 2023; Kramer *et al.*, 2013; Rudd *et al.*, 2012; Yaden *et al.*, 2019). Self-loss reflects the phenomenon where awe can diminish one’s sense of self, leading to feelings of insignificance ( Bai *et al.*, 2017; Piff *et al.*, 2015; Takano & Nomura, 2021). Connectedness indicates that awe experiences can create a greater sense of connection between individuals and their surroundings, which includes the supernatural, such as God(s) ( Liu *et al.*, 2023; Van Cappellen & Saroglou, 2012). Vastness pertains to the perception of vastness vis-à-vis the self, where individuals encounter physically or conceptually enormous stimuli ( Keltner & Haidt, 2003; Landrum *et al.*, 2022). Physiological captures awe-related physiological phenomena, such as goosebumps ( Quesnel & Riecke, 2018; Williams *et al.*, 2022). Accommodation reflects the process of needing to adjust one’s understanding or cognitive framework to comprehend new information or experiences, such as those often experienced during awe-inspiring situations ( Keltner & Haidt, 2003; Taylor & Uchida, 2019; Valdesolo *et al.*, 2016). Yaden *et al.* (2019) showed that the AWE-S, measured after one recalled and described their experience of awe, was positively associated with trait measures of tendency to experience awe, which was salient for the factors of time, connectedness, vastness, and physiological. This suggested that the state measure of awe had enough convergent and divergent validity.

Due to its robustness and usefulness, the AWE-S has been validated in several countries and cultures via translation into other languages, such as Chinese, Portuguese, and Dutch ( Landrum *et al.*, 2022; Li & Qian, 2022; Ling-xiao *et al.*, 2022; Rodrigues *et al.*, 2022; van Houwelingen-Snippe *et al.*, 2020). Universal components and cultural variations of awe-related phenomena have been identified via this scale. However, despite the creation and development of other scales to measure awe in Japan, the AWE-S has not yet been translated into the Japanese language ( Nakayama *et al.*, 2020; Sawada & Nomura, 2023). To facilitate further comprehensive cross-cultural comparisons, development of a Japanese version of the AWE-S, which can provide a deeper understanding of awe, is necessary.

Previous studies suggested that cultural similarities and differences in awe experiences were important perspectives to understand its generality and heterogeneity ( Bai *et al.*, 2017; Nakayama *et al.*, 2020; Nakayama & Uchida, 2020; Razavi *et al.*, 2016; Sawada & Nomura, 2023). Specifically, it was necessary to consider how awe altered the sense of self and differed across cultures, although the small self-effect was a general and robust phenomenon ( Bai *et al.*, 2017; Nomura *et al.*, 2021; Takano & Nomura, 2021). Awe could manifest differently among individuals from East Asian cultures, such as Japan, compared to those from Western cultures according to their culturally, historically, and religiously cultivated views of the self. Particularly, the self is represented as being non-substantial, denial, and embedded as default in Japan, a country with Buddhist and Shinto backgrounds. Meanwhile, it is represented as separated and continuous concepts by others in Western cultures ( Nomura *et al.*, 2021; Sugimoto, 2020; Takano & Nomura, 2019, 2022a). Similarly, Markus and Kitayama (1991) advocated independent and interdependent self-construals. Independent self-construals, more widespread in North American and Western European cultures, prioritize an individual’s needs, whereas interdependent self-construals, more common in Asian, Latin American, and African cultures, prioritize one’s social interactions. Thus, a Japanese AWE-S could reveal culture-specific patterns, especially regarding sense of self.

From the perspective of state awe, it is important to address how people narrate their awe experiences in their cultural context for a deeper understanding ( Nakayama & Uchida, 2020). Since the AWE-S is measured after participants recall and describe (i.e., write) their awe experiences ( Yaden *et al.*, 2019), their contents may have a role manifesting the responses to the AWE-S. Studies revealed that ratings on the AWE-S varied based on the elicitors of awe experiences ( Graziosi & Yaden, 2021; Yaden *et al.*, 2019). However, the aspects of narrated experience (i.e., the “potential topics”) associated with each factor of the AWE-S have not been investigated beyond the typology of awe experience. For example, looking up at the night sky or touching a grand theory are different types of experiences. However, both could be accompanied with common potential topic of “universe.” Similarly, different awe experiences narrated through the same elicitor could involve distinct potential topics, which could lead to the AWE-S being rated differently.

This study aimed to develop a standardized Japanese AWE-S as well as investigate its relationships with linguistic generative processes underlying the expression of awe experiences. First, we created a Japanese version of the AWE-S via back-translation and confirmed its factor structure and test-retest reliability compared to the original AWE-S. Furthermore, its convergent and divergent validities were examined regarding its relation to the trait-awe (dispositional awe) questionnaire. We used the Dispositional Positive Emotion Scale (DPES) since previous studies demonstrated that awe-related phenomena observed via the DPES could also be seen through the measurement of state-awe ( Bai *et al.*, 2017; Piff *et al.*, 2015; Stellar *et al.*, 2018). We controlled other positive emotions to examine specific relationships between dispositional awe and each factor of the Japanese AWE-S. Second, we used a structural topic model (STM) and exploratorily examined whether and how each factor of AWE-S was associated with topics that underlie the narrative of awe experiences ( Roberts *et al.*, 2014). Using this model, we can focus not only on the *type* of experience, but also on the generative process of potential topics underlying how people feel in that experience and express it in natural language.

## Methods

### Study design and participants

This study recruited Japanese participants through Qualtrics ( http://www.qualtrics.com) via a Japanese crowdsourcing service, Crowd Works ( https://crowdworks.jp/). Participants were required to meet the a priori criterion that they were native Japanese speakers. For the main study, 358 responses were collected in November 2022. After 43 participants with incorrect answers for attention checks or duplicated IP addresses were excluded, 315 respondents were included (156 males, 156 females, and three others, *M _age_* = 42.64 years, *SD* = 10.08, *Range*: 20–79 years). Our target sample size was determined via a priori power analysis (G*Power) ( Faul *et al.*, 2007). To achieve 0.90 power at an α level of 0.05 for an effect size of *r* = .20, a medium effect of some explanatory and practical use even in the short run, a sample of 255 participants was required ( Funder & Ozer, 2019). To evaluate the test-retest reliability of the Japanese AWE-S, we requested participants to complete the same questionnaire again approximately one week later ( *N* = 91, 42 males, 48 females, and one other, *M _age_* = 43.37 years, *SD* = 9.86, *Range*: 24–71 years). Additionally, before data were collected for the main study, we conducted a separate pilot survey to assess the mean and variance of each item in the Japanese version of the AWE-S ( *N* = 137, 66 males, 70 females, and one other, *M _age_* = 42.26 years, *SD* = 8.78, *Range*: 22–64 years). Since the items used in the pilot survey were identical to those in the main study, we conducted factor analyses of the Japanese version of the AWE-S and text analyses via a combined sample of participants from both the pilot and main study ( *N* = 452). The questionnaires took approximately 15–20 minutes to answer and participants were paid 200 yen for their time (regarding the additional survey for the test-retest reliability, we paid 50 yen for 5 minutes).

Table 1 presents the participants’ demographic information in the main and pilot studies as well as the statistical analyses.

**Table 1.** Participants’ demographic information and applied statistical analyses in the main and pilot studies.

	Main study	Pilot study
*N*	315	137
Gender		
Males	156	66
Females	156	70
Others	3	1
Mean age	42.64 years	42.26 years
Statistical analysis		
Factor analysis of the AWE-S	X	X
Factor analysis of the DPES	X	
Test re-test reliability	X ( *N* = 91)	
*T*-test	X	X
Correlation and regression analysis	X	
Text analysis	X	X

*Note*. X denotes that the statistical analysis was applied. AWE-S = Awe Experience Scale, DPES = Dispositional Positive Emotion Scale.

The study conformed to the principles expressed in the Declaration of Helsinki and its future amendments. This study was approved by the Ethics Committee of Tokaigakuen University (Ref-No. 2022-11). Written informed consent was obtained from all the study participants at the start of the survey. Participants had the right to withdraw from the experiment at any time without providing a reason. They were also explained to them that their responses would not be tied to them personally. This study was partly preregistered at https://aspredicted.org/n6ga7.pdf. Data and analysis codes are available under *Underlying data* ( Takano & Matsuo, 2023b).

### Measures

The following measures were used (we assessed other questionnaires for other related investigations), and participants’ demographic information (gender, age, and nationality) were enquired.

Japanese versions of the Awe Experience Scale

We translated the instructions and items of the AWE-S into Japanese with permission from the original authors. Subsequently, we used a translation service (NAI Inc.; https://www.nai.co.jp/) to back-translate the provisional Japanese version. Specific instructions in Japanese are available under *Extended data* ( Takano & Matsuo, 2023b).

As per Yaden *et al.*’s study (2019), participants were instructed to recall a recent and intense awe experience and write it. Specifically, the instructions asked participants to reflect on a specific moment in time when they felt intense awe, and describe that experience in approximately two paragraphs in a designated text box. The instructions emphasized that participants should focus on the experience itself rather than what led up to it, what happened afterwards, or their interpretation. In addition, they were required to be as descriptive and specific as possible. Subsequently, participants responded to 30 items on the aspects of their awe experience. Each item was rated on a 7-point scale (1 = *Strongly Disagree*, 7 = *Strongly Agree*). To prevent the effects of recalling an awe experience on responses to other questionnaires, participants completed the AWE-S after all the other questionnaires.

Dispositional Positive Emotion Scale (DPES)

The Dispositional Positive Emotion Scale (DPES), a trait-based assessment tool that measured an individual’s tendency to experience various positive emotions in daily life, was used to examine the construct validity of the Japanese version of the AWE-S. The scale consisted of 38 items that included several subscales, which included joy (six items, α = .86), amusement (five items, α = .78), awe (six items, α = .80), contentment (five items, α = .95), love (six items, α = .86), pride (five items, α = .81), and compassion (five items, α = .83). The original version of this scale was developed by Shiota *et al.* (2006). We used the Japanese version developed by Nomura *et al.* (2021).

### Statistical analyses

Data analyses were conducted using R software (version 4.2.2 [2022-10-31]). First, we conducted confirmatory factor analyses for the DPES and Japanese AWE-S via lavaan SEM package ( Rosseel, 2012). Second, to examine the test-retest reliability of the Japanese AWE-S, we calculated the intraclass correlation coefficients between the first and second responses for each factor using the irr package ( Gamer *et al.*, 2012). Third, we used an independent *t*-test to compare the means of the Japanese AWE-S factors with those of the original AWE-S factors ( Yaden *et al.*, 2019). Fourth, zero-order correlation analyses were conducted to assess the relationships among the subfactors of these scales. Fifth, linear regression analyses were performed with a robust estimation method to investigate whether the Japanese AWE-S were associated with the DPES awe factor, controlled for other DPES factors, using the ‘lm_robust’ function in the estimatr package ( Blair *et al.*, 2021). For the regression analyses, all (in) dependent variables were standardized.

For the text analyses, participants’ descriptions of their awe experiences were analyzed via the stm package to estimate a Structural Topic Model ( Roberts *et al.*, 2019, 2014). We investigated how the AWE-S was associated with their awe narratives (note that these analyses were exploratory and not pre-registered). This method considered additional information regarding the data structure (i.e., the AWE-S in this study), which was incorporated into the model to help identify and extract more meaningful and interpretable topics. We added six factors of the AWE-S as covariates to examine how the prevalence of each topic changed when each factor rating increased or decreased and whether the change was significant.

Descriptions of the awe experiences underwent tokenization and pre-processing based on Roberts *et al.*’s study (2014) ( *N* = 452). We regarded each participant’s description as one document. Tokens were limited to adjectives, verbs, and nouns that occurred at least three times across all the participants. Additionally, we used the ‘prepDocuments’ function in the stm package and eliminated numbers, common punctuation, and stop-words considered semantically meaningless. The number of topics ( *K*) was determined using the ‘search *K*’ function by generating models based on potential *K*s that ranged from 2 to 50. The model with *K* = 7 had a sufficient held-out log-likelihood and average semantic coherence across topics, whereas these indices dropped drastically when *K*s were over 20. Therefore, we selected *K* = 7. After the STM was estimated, each author independently assigned labels based on the most frequent words listed in each topic. We discussed these until a consensus was reached on a label for the topic ( Idler *et al.*, 2022; Sterling *et al.*, 2019).

## Results

A total of 452 participants (222 males, 226 females, and four others, *M _age_* = 42.52 years, *SD* = 9.69, *Range*: 20–79 years) were included in the factor analysis, *t*-tests, and the STM for the Japanese AWE-S ( Table 1). Of these, 91 participants (42 males, 48 females, and one other, *M _age_* = 43.37 years, *SD* = 9.86, *Range*: 24–71 years) were included in the intra-class correlation analyses to examine the test-retest reliability. In addition, 315 participants (156 males, 156 females, and three others, *M _age_* = 42.64 years, *SD* = 10.08, *Range*: 20–79 years) were included in the factor analysis for the DPES, correlation analyses, and multiple regression analyses. The full dataset can be found under *Underlying data* ( Takano & Matsuo, 2023b).

Confirmatory factor analyses were conducted to examine the factor structure of the Japanese AWE-S ( Table 2) and DPES. The results showed that the six-factor model provided an adequate fit to the data for the Japanese AWE-S, chi-squared (χ ^2^) (390) = 1070.31, Comparative Fit Index (CFI) = .905, Goodness Fit Index (GFI) = .854, Root Mean Square Error of Approximation (RMSEA) = .062 [90% lower = .058], and Standardized Root Mean Square Residual (SRMR) = 0.068. In addition, the seven-factor model of the DPES fitted well as in previous studies, χ ^2^ (644) = 1728.55, CFI = .860, GFI = .763, RMSEA = .073 [90% lower = .069], and SRMR = .064 ( Nomura *et al.*, 2021; Shiota *et al.*, 2006; Sugawara *et al.*, 2020). To examine the internal consistency of the Japanese AWE-S, Cronbach’s alpha coefficients were calculated for each subscale ( Table 2). Sufficient internal consistency was confirmed, which was consistent with the results of previous studies ( Ling-xiao *et al.*, 2022; Yaden *et al.*, 2019). In addition, the intraclass correlations analyses for each subscale of AWE-S showed sufficient and good test-retest reliability (ρs > .60, *p* < .001, Table 2).

**Table 2.** Items in the Japanese version of the Awe Experience Scale and results of the confirmatory factor analysis.

	Items	Factor Loadings	*Mean*	*SD*
Time (α = .88, ρ = .76, *M* = 4.83, *M*_Yaden *et al.* (2019)_ = 4.83)				
1	I sensed things momentarily slow down.	.80	4.54	1.63
	その瞬間、あらゆるものがゆっくりと動いているように感じた。			
2	I noticed time slowing.	.81	4.58	1.53
	気がつくと時間がゆっくり進んでいた。			
3	I felt my sense of time change.	.77	4.69	1.55
	時間の感覚が変化しているように感じた。			
4	I experienced the passage of time differently.	.76	5.37	1.38
	普段とちがう時間の流れを経験した。			
5	I had the sense that a moment lasted longer than usual.	.75	4.98	1.48
	普段よりも一瞬が長く感じられた。			
Self-loss (α = .83, ρ = .72, *M* = 4.76, *M*_Yaden *et al.* (2019)_ = 4.35)				
6	I felt that my sense of self was diminished.	.72	4.37	1.52
	自分という感覚が薄れていくように感じた。			
7	I felt my sense of self shrink.	.75	4.62	1.52
	自分という感覚が縮むように感じた。			
8	I experienced a reduced sense of self.	.70	4.26	1.56
	自分という感覚が低下するように感じた。			
9	I felt my sense of self become somehow smaller.	.72	5.11	1.47
	自分という感覚がなにか小さくなっていくように感じた。			
10	I felt small compared to everything else.	.63	5.42	1.47
	他のすべてのものと比べて自分を小さく感じた。			
Connectedness (α = .92, ρ = .79, *M* = 3.96, *M*_Yaden *et al.* (2019)_ = 4.99)				
11	I had the sense of being connected to everything.	.85	4.20	1.66
	すべてのものと繋がっているように感じた。			
12	I felt a sense of communion with all living things.	.83	3.71	1.66
	あらゆる生き物と一体になっている感覚があった。			
13	I experienced a sense of oneness with all things.	.86	4.05	1.65
	すべてのものとの一体感を経験した。			
14	I felt closely connected to humanity.	.76	3.85	1.72
	人類全体と密接に繋がっているように感じた。			
15	I had a sense of complete connectedness.	.83	3.97	1.62
	完全に繋がっている感覚があった。			
Vastness (α = .87, ρ = .65, *M* = 5.97, *M*_Yaden *et al.* (2019)_ = 5.63)				
16	I felt that I was in the presence of something grand.	.78	5.93	1.23
	何か雄大な存在を目の当たりにしているように感じた。			
17	I experienced something greater than myself.	.83	6.01	1.26
	自分よりも広大な存在を経験した。			
18	I felt in the presence of greatness.	.66	5.84	1.30
	偉大なものの存在を感じた。			
19	I perceived something that was much larger than me.	.78	6.21	1.13
	自分よりはるかに大きな存在を感じた。			
20	I perceived vastness.	.74	5.87	1.44
	広大さを感じた。			
Physiological (α = .81, ρ = .60, *M* = 4.85, *M*_Yaden *et al.* (2019)_ = 5.02)				
21	I felt my jaw drop.	.69	4.61	1.61
	思わず口が開いてしまうのを感じた。			
22	I had goosebumps.	.76	4.65	1.66
	鳥肌がたった。			
23	I gasped.	.68	5.43	1.34
	息を呑んだ。			
24	I had chills.	.67	4.66	1.70
	背筋がゾクゾクするような感覚があった。			
25	I felt my eyes widen.	.61	4.91	1.54
	目が見開くのを感じた。			
Accommodation (α = .82, ρ = .67, *M* = 4.29, *M*_Yaden *et al.* (2019)_ = 4.76)				
26	I felt challenged to mentally process what I was experiencing.	.79	4.24	1.57
	自分が経験していることを頭の中で処理できないように感じた。			
27	I found it hard to comprehend the experience in full.	.81	4.32	1.62
	自分が経験していることを完全に理解することは難しいと思った。			
28	I felt challenged to understand the experience.	.81	3.92	1.66
	その経験を理解することは難しいと感じた。			
29	I struggled to take in all that I was experiencing at once.	.73	4.12	1.52
	自分が経験していることを一度にすべて受けとめることがなかなかできなかった。			
30	I tried to understand the magnitude of what I was experiencing.	.31	4.85	1.37
	自分が経験していることの大きさを理解しようとした。			

*Note.* *N* = 452 (91 for intraclass correlation coefficients), α = Cronbach's coefficient alpha, ρ = intraclass correlation coefficient.

There were differences in the means of some factors of the AWE-S between the Japanese and original versions, as shown in Table 2. Independent *t*-tests indicated that the mean scores for the self-loss and vastness factors were higher in the Japanese version than in the original. In contrast, those for the connectedness, physiological and accommodation factors were lower in the Japanese version than in the original (self-loss: *t* (1086) = 4.90, *p* < .001, vastness: *t* (1086) = 4.97, *p* < .001, connectedness: *t* (1086) = -11.66, *p* < .001, and physiological: *t* (1086) = -2.20, *p* = .028, accommodation: *t* (1086) = -5.99, *p* < .001).

Correlation analyses between the subscales of the Japanese AWE-S and DPES were conducted to examine construct validity. Time, connectedness, vastness, and physiological factors were significantly positively correlated with awe ( *r* = .12–.36, *p* < .038) and other factors of the DPES; however, self-loss and accommodation were not ( Table 3). In addition, while the inter-factor correlations coefficients of the Japanese AWE-S ranged from moderate to large, those of the DPES were large (AWE-S: *r* = .26–.47, *p* < .001, DPES: *r* = .44–.83, *p* < .001).

**Table 3.** Results of the correlation and linear regression analyses with a robust estimation predicting each factor of the Japanese version of Awe Experience Scale from that of the Dispositional Positive Emotion Scale.

		Japanese version of the AWE-S											
		Time		Self-loss		Connectedness		Vastness		Physiological		Accommodation	
		*r*	β	*r*	β	*r*	β	*r*	β	*r*	β	*r*	β
DPES	Awe	.34 *	.21 *	.08	.07	.36 *	.23 *	.12 *	-.02	.18 *	.04	.07	-.03
	Amusement	.25 *	.00	.04	.02	.24 *	-.03	.04	-.18 *	.16 *	.02	.08	.04
	Joy	.32 *	.16	.02	-.13	.32 *	.10	.20 *	.37 *	.21 *	.30 *	.08	.02
	Compassion	.27 *	.08	.11	.10	.30 *	.11	.19 *	.12	.22 *	.12	.16 *	.15
	Love	.25 *	-.02	.08	.08	.25 *	-.07	.10	-.12	.14 *	-.02	.10	.04
	Pride	.26 *	-.00	.06	.09	.32 *	.20 *	.17 *	.14	.13 *	-.01	.09	.07
	Contentment	.27 *	.00	.01	-.11	.26 *	-.10	.12 *	-.14	.10	-.22	.04	-.14

*Note. N* = 315, AWE-S = Awe Experience Scale, DPES = Dispositional Positive Emotion Scale.

^*^ = *p* < .05.

Linear regression analyses with a robust estimation method showed that the time and connectedness factors were significantly positively associated with awe factor of the DPES, controlled for other positive emotions (β = .21–.23, *p* < .013). This indicated that these factors of the AWE-S were specifically associated with dispositional awe. These results did not change when controlled for participants’ age and gender.

**Figure 1.**
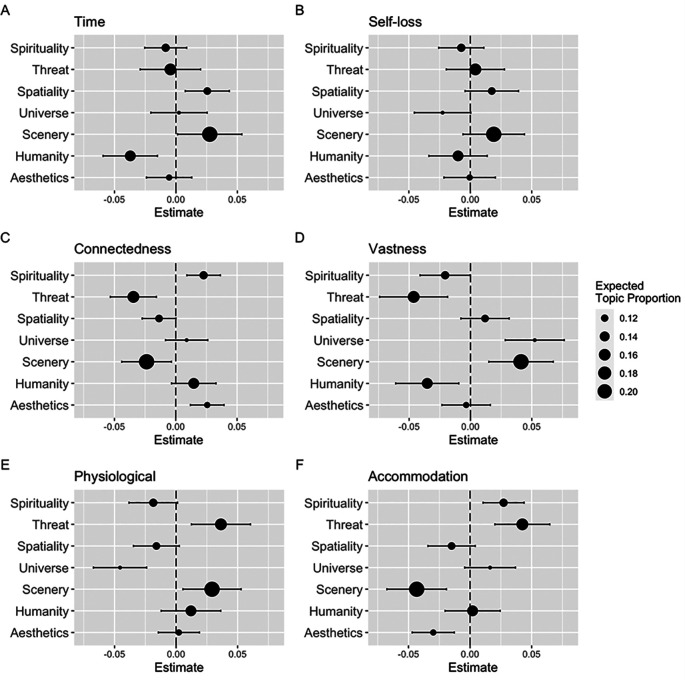
Parameter estimate of the effect of each factor of the Japanese Awe Experience Scale on the seven topics. *Note.* Effects computed via a structural topic model. Parameter estimates are shown as dots. Expected topic proportions, proportion of the total documents (responses) falling into each topic, are depicted by dot size. Error bars represent 95% confidence interval.

Furthermore, seven topics were generated by the STM and labeled: “Spirituality,” “Threat,” “Spatiality,” “Universe,” “Scenery,” “Humanity,” and “Aesthetics” (see Figure 1). The top 20 highest probability words are listed in Table 4. In particular, “Scenery” and “Threat” were the two most common topics that emerged in all the responses. Regarding the Japanese AWE-S, participants with higher scores on the time factor were more likely to use words related to “Spatiality” and “Scenery” ( Figure 1A). In contrast, they were less likely to use words of “Humanity.” Similarly, positive associations were found between the connectedness factor and “Spirituality” and “Aesthetics” ( Figure 1C), vastness factor and “Universe” and “Scenery” ( Figure 1D), physiological factor and “Threat” and “Scenery” ( Figure 1E), and accommodation factor and “Spirituality” and “Threat” ( Figure 1F). In addition, negative associations were found between the connectedness factor and “Threat” and “Scenery” ( Figure 1C), vastness factor and “Spirituality,” “Threat,” and “Humanity” ( Figure 1D), physiological factor and “Universe” ( Figure 1E), and accommodation factor and “Scenery” and “Aesthetics” ( Figure 1F). Self-loss was not significantly associated with any topic ( Figure 1B).

**Table 4.** Top words for Awe-Experience topics.

Topic	Top 20 highest probability word list
Spirituality	self, can, feelings, being, shrine, now, mind, wonder, living, atmosphere, place, understanding, Japan, world, temple, opportunity, know, sense, time, body
	自分, できる, 気持ち, 存在, 神社, 今, 心, 不思議, 生きる, 感じ, 場所, わかる, 日本, 世界, お寺, 機会, 知る, 感覚, 時間, 体
Threat	*ifu*, strong, earthquake, typhoon, fear, afraid, home, force, experience, before, can, river, near, disaster, this year, scary, wind, big, news, region
	畏怖, 強い, 地震, 台風, 恐怖, 怖い, 家, 力, 経験, 前, できる, 川, 近く, 災害, 今年, 恐ろしい, 風, 大きい, ニュース, 地域
Spatiality	seeing, sky, stars, trees, looking up, big, starry sky, clouds, vast, eyes, senses, air, night, view, spreading, beautiful, experience, moment, self, mountain top
	見える, 空, 星, 木, 見上げる, 大きい, 星空, 雲, 広い, 目, 感覚, 空気, 夜, 様子, 広がる, 綺麗, 体験, 瞬間, 自分, 山頂
Universe	moon, cosmos, lunar eclipse, total, earth, can, mysterious, other day, celestial, usual, vast, wane, different, human, Uranus, planet, observe, outside, inspiring, happening
	月, 宇宙, 月食, 皆既, 地球, できる, 神秘, 先日, 天体, 普段, 壮大, 欠ける, 違う, 人間, 天王星, 惑星, 観察, 外, 感動, 起こる
Scenery	going, landscape, mountain, sea, travel, waterfall, front, walking, climbing, small, self, inspiring, overwhelming, strong, top, magnificent, place, climbing, can, being
	行く, 景色, 山, 海, 旅行, 滝, 前, 歩く, 登山, 小さい, 自分, 感動, 圧倒, 強い, 頂上, 雄大, 場所, 登る, できる, 存在
Humanity	human, thinking, being, self, sense, having, die, great, oneself, born, age, can, know, feet, video, children, appearance, respect, coming, atmosphere
	人, 思う, いる, 自分, 念, 持つ, 亡くなる, すごい, 自身, 生まれる, 時代, できる, 知る, 足, 動画, 子ども, 姿, 尊敬, 来る, 気
Aesthetics	sense, beautiful, human, remember, out, have, light, trees, sun, life, great, circumstances, other day, early, autumn leaves, walk, wonderful, mood, morning, run
	念, 美しい, 人間, 覚える, 出る, 抱く, 光, 木々, 太陽, 命, 偉大, 状況, 先日, 早い, 紅葉, 散歩, 素晴らしい, 気分, 朝, 走る

*Note.* The authors translated Japanese words into English.

## Discussion

This study aimed to develop a Japanese version of the AWE-S, a robust questionnaire that measured the subjective state in awe experiences, and investigate its relations to the representations of awe experiences in natural language. We examined the validity of the Japanese AWE-S and explored how each factor of the AWE-S was related to topics that underlie the narrative of awe experiences via a structural topic model. The results showed that the six-factor model of the original AWE-S sufficiently fit the data for the Japanese AWE-S. Furthermore, the mean scores for the self-loss and vastness factors were higher in the Japanese version than in the original. In contrast, the connectedness, physiological and accommodation factors were lower in the Japanese version. In addition, the time, connectedness, vastness, and physiological factors were positively associated with trait-awe (dispositional awe). The relationships with the time and connectedness factors remained constant, even when controlled for other positive emotions, age, and gender. The STM generated seven potential topics, which were differently associated with each factor of the Japanese AWE-S.

The confirmatory factor analysis revealed that the Japanese AWE-S conformed to the six-factor model of the original ( Yaden *et al.*, 2019). In addition, Cronbach’s alpha coefficients and intraclass correlations showed that this scale had sufficient internal consistency and test-retest reliability, respectively. Therefore, our results suggest that the structure and reliability of the Japanese AWE-S are comparable to those of the original version, which rendered it suitable for international comparisons.

The time, connectedness, vastness, and physiological factors of the Japanese AWE-S were positively associated with trait measures of tendency to experience awe (i.e., dispositional awe). These results were consistent with Yaden *et al.*’s results (2019), which suggested that the Japanese version had similar construct validity to the original. Furthermore, we used linear regression analyses and revealed that the time and connectedness factors were specifically related to trait-awe, controlled for other positive emotions. Previous studies suggested that the dispositional awe questionnaire mainly focused on the positivity, beauty, and mindful aspects of awe (example: “I see beauty all around me” ( Shiota *et al.*, 2006). Therefore, our results suggested that the AWE-S was a state scale that could capture the other aspects of awe.

Regarding the culturally specific perspectives, the mean scores for the self-loss and vastness factors were higher, while those for the connectedness, physiological and accommodation factors were lower in the Japanese version than in the original. Previous research suggested that the sense of the self was mainly characterized by being non-substantial, denied, and embedded as default in Japan with its Buddhist and Shinto backgrounds, while it is represented as separated and continuous concepts by others in Western cultures ( Nomura *et al.*, 2021; Sugimoto, 2020; Takano & Nomura, 2021, 2022a). Consistent with this perspective, Takano and Nomura (2021) revealed that experimentally induced awe reduced one’s sense of self-size and also blurred the sense of boundary of the self. Hence, Japanese people might be more and less likely to respond to self-reductive (i.e., self-loss and vastness) and self-expansive aspects during awe experiences (i.e., connectedness, physiological, and accommodation), respectively.

Regarding the narrative aspect of awe, the STM revealed that each factor of the AWE-S was related to seven topics of descriptions of awe experiences. There were positive (negative) associations between the time factor and “Scenery” and “Spatiality” (“Humanity”), connectedness factor and “Spirituality” and “Aesthetics” (“Threat” and “Scenery”), vastness factor and “Universe” and “Scenery” (“Spirituality,” “Threat,” and “Humanity”), physiological factor and “Threat” and “Scenery” (“Universe”), and accommodation factor and “Spirituality” and “Threat” (“Scenery” and “Aesthetics”). These results were consistent with the characteristics of each factor of the original AWE-S ( Yaden *et al.*, 2019). Previous studies also demonstrated that the elicitors of awe experiences had different roles in manifesting responses to the AWE-S ( Graziosi & Yaden, 2021; Yaden *et al.*, 2019). Therefore, beyond the typology, this was the first study to reveal that the various psychological elements of awe emerged differently based on the potential topics of the narratives.

It should be also noted that “Scenery” was the most common topic, which was positively related to the vastness factor of the Japanese AWE-S. This topic mainly consisted of nature-related words, such as “landscape,” “mountain,” and “sea.” Previous studies have demonstrated that many Japanese people are familiar with nature worship, a belief that the self is a part of deified nature that is cultivated under the syncretism of Shinto and Buddhism ( Sugimoto, 2020; Takano & Nomura, 2022a). Therefore, these results might reflect the Japanese view of the self in relation to nature worship (i.e., non-substantial, denial, embedded).

Interestingly, “Threat,” the second common topic, might be associated with negative aspects of awe (i.e., threat-awe), which is in line with the fact that natural disasters occur frequently in Japan due to its climate and topography. Previous studies suggested that feelings of awe that are triggered by threatening stimuli such as natural disasters could be tinged with the perception of threat ( Takano & Nomura, 2022b). Given that even the recollection of awe experiences based on examples that do not include negative elements (e.g., seeing aurora) evokes the feelings of *ifu*, negative awe in Japanese ( Takano & Nomura, 2023a), Japanese people might be more likely to feel threatened during awe experiences due to Japan’s geographic and historical background.

This study has some limitations and directions for future research. First, along with the original version, the effect sizes of the correlations between some of the factors of the AWE-S and trait-awe were relatively low ( Yaden *et al.*, 2019). This may have been caused by ceiling effects, which should be investigated in future studies. Second, when writing regarding previous experiences, it was possible that the participants’ ratings were influenced by their memory of the event and not the actual experience of awe. The recollection method allowed us to investigate the narrative dimensions of the AWE-S. As in a previous study ( Bai *et al.*, 2017; Valdesolo & Graham, 2014; Van Cappellen & Saroglou, 2012), other manipulation methods (e.g., video stimuli) should be used to confirm the generalizability of our results. Third, this study investigated the relationship between the Japanese AWE-S and self-reported measures to reveal its construct validity. Given that the advantage of the AWE-S was that it revealed psychological processes that could only be captured subjectively, further studies should investigate whether and how this scale was associated with other behavioral, physiological, and neural measures.

In summary, this study is the first to develop a Japanese version of the AWE-S, confirm its convergent and divergent validities, and investigate its narrative dimensions during awe experiences. Our findings contribute to a deeper understanding of awe and revealing its construct in Japan through cross-cultural comparisons. Furthermore, this study provides conceptual and methodological implications regarding studies on awe.

## Data Availability

OSF: Japanese ver. of AWE-S and Purity. https://doi.org/10.17605/OSF.IO/8CSR4 ( Takano & Matsuo, 2023b). This project contains the following underlying data:
- data_cleaned.csv (dataset for the main study) -
data_pilot_cleaned.csv (dataset for the pilot study) - data_testretest.csv (dataset for the test-retest validity) - instructionsforJverAWE-S.docx (specific instructions for the Japanese AWE-S) - main_script.R (analysis code) OSF: Japanese ver. of AWE-S and Purity. https://doi.org/10.17605/OSF.IO/8CSR4 ( Takano & Matsuo, 2023b). This project contains the following extended data:
-
questionnaire_in_Japanese.pdf Data are available under the terms of the Creative Commons Attribution 4.0 International license (CC-BY 4.0).
