# Supplementary material for: Development of a Japanese version of the Awe Experience Scale (AWE-S): A structural topic modeling approach
Source: F1000Res. 2023 Oct 13;12:515. Originally published 2023 May 18. [Version 2] doi: 10.12688/f1000research.134275.2 (PMC10611950; doi:10.12688/f1000research.134275.2)
Supplement: Peer review history

# Reviewer report: Reviewer response for version 2

**DOI:** 10.5256/f1000research.157333.r215152

**Published:** 2023-10-27

**Authors:** Hidefumi Hitokoto

**Affiliations:** 1 School & Graduate School of Humanities, Kwansei Gakuin University, Nishinomiya, Japan

Thank you for your thorough response and/or explanation.

 Since all of my previous concerns have been addressed, I agree to accept the manuscript as lastly submitted. With these details, I'm sure the J-AWE-S is initially validated, and I hope the best for the authors to further strengthen the scale validity in their following studies, across cultures.

Is the work clearly and accurately presented and does it cite the current literature?

Yes

If applicable, is the statistical analysis and its interpretation appropriate?

I cannot comment. A qualified statistician is required.

Are all the source data underlying the results available to ensure full reproducibility?

Partly

Is the study design appropriate and is the work technically sound?

Partly

Are the conclusions drawn adequately supported by the results?

Partly

Are sufficient details of methods and analysis provided to allow replication by others?

Partly

Reviewer Expertise:

NA

I confirm that I have read this submission and believe that I have an appropriate level of expertise to confirm that it is of an acceptable scientific standard.

# Reviewer report: Reviewer response for version 1

**DOI:** 10.5256/f1000research.147318.r207846

**Published:** 2023-09-28

**Authors:** William L. D. Krenzer

**Affiliations:** 1 Duke University, Durham, North Carolina, USA

The authors for this paper set out to create a Japanese version of the AWE-S scale previously published (Yaden et al., 2019). Following the steps of previous researcher translating AWE-S, the researchers in this article went forward with translating the AWE-S into Japanese and getting the appropriate population to complete the survey in order to validate the translation.

 The researchers conducted a fairly conservative power analysis for determining the proper sample size needed, as well as setting an a prori criteria for including participants.

 Overall, i think that the researchers did a great job with this manuscript and conducting this study. I think that they have all the relevant citations, the study design is sound, and the researchers did a great job with addressing the findings that were specific to their Japanese participants.

 There is one main recommendation to the researchers to enhance the clarity of the paper:

- In the study design and participant section they do a great job of describing the main sample (N = 315) but rush through the other two samples that they use, and it makes it hard to really understand who these people are and why exactly they are being used and how they are different.

I think that this manuscript will be a great addition to the awe literature and psychology as a whole.

Is the work clearly and accurately presented and does it cite the current literature?

Yes

If applicable, is the statistical analysis and its interpretation appropriate?

Yes

Are all the source data underlying the results available to ensure full reproducibility?

Yes

Is the study design appropriate and is the work technically sound?

Yes

Are the conclusions drawn adequately supported by the results?

Yes

Are sufficient details of methods and analysis provided to allow replication by others?

Yes

Reviewer Expertise:

Research integrity, open science practices, awe, facial recognition

I confirm that I have read this submission and believe that I have an appropriate level of expertise to confirm that it is of an acceptable scientific standard.

# Response

**Published:** 2023-10-10

**Authors:** Ryota Takano

**Affiliations:** Social Psychology, The University of Tokyo, Bunkyo-ku, Tokyo, Japan

**Response to Reviewer #2:**

 The authors for this paper set out to create a Japanese version of the AWE-S scale previously published (Yaden et al., 2019). Following the steps of previous researcher translating AWE-S, the researchers in this article went forward with translating the AWE-S into Japanese and getting the appropriate population to complete the survey in order to validate the translation.

 The researchers conducted a fairly conservative power analysis for determining the proper sample size needed, as well as setting an a prori criteria for including participants.

 Overall, i think that the researchers did a great job with this manuscript and conducting this study. I think that they have all the relevant citations, the study design is sound, and the researchers did a great job with addressing the findings that were specific to their Japanese participants.

**Reply**: Thank you for taking the time and effort to check our manuscript. We appreciate your helpful comment. We incorporated all your comments in the manuscript. Point-by-point responses and the corresponding revised text are provided below.

**Comment 1.**

 There is one main recommendation to the researchers to enhance the clarity of the paper:

- In the study design and participant section they do a great job of describing the main sample ( *N* = 315) but rush through the other two samples that they use, and it makes it hard to really understand who these people are and why exactly they are being used and how they are different.

**Reply**: Thank you for your suggestion. According to your comment, we have summarized the demographic information of samples in the main and pilot study, along with the respective statistical analyses applied, in Table 1.

# Reviewer report: Reviewer response for version 1

**DOI:** 10.5256/f1000research.147318.r174203

**Published:** 2023-06-19

**Authors:** Hidefumi Hitokoto

**Affiliations:** 1 School & Graduate School of Humanities, Kwansei Gakuin University, Nishinomiya, Japan

I enjoyed reading the manuscript a lot. As it is deals with the cultural generalization of the AWE experience measure, especially in a culture where previous studies showed some interesting cultural differences between the West (Nakayama et al., 2020), the scale standardization should contribute to the test of universality of the emotion.

 My comments are listed below. They are mostly on the logic building up to the interpretation of the main results.

 I understand the authors are trying to make the case that the newly translated J-AWE-S has reliability and validity, including factorial and criterion-related. However, the current unfolding seems to make an impression that the correlations with DPES are given post-hoc interpretations. In other words, how can the validity correlations with DPES (or the lack thereof with self-loss and accommodation) be understood as supporting the validity of the J-AWE-S? Similarly, what was the logic behind controlling other positive emotions of DPES to arrive at the regression result of selected positive emotions explaining seemingly different aspects of J-AWE-S as in Table 2?

 Regarding J-AWE-S, Item 30 seems to show low factor loading. Would removing it contribute to the improvement of the fit?

 DPES seems to show a marginal fit. Does it reliably measure the 7 positive emotions? Or, removing some items/emotions increases the overall reliability, and that makes a better test?

 As the text analysis of the experience topic and its correspondence with J-AWE-S response is so interesting, if the main aim of the study is to validate the scale in conjunction with the former, authors may present some expected findings given the nature of the J-AWE-S. This seemed possible a-pri-ori, when authors argued that awe involves vastness in scenes or has cultural roots of selfhood.

 How do we make of the "Expected Topic Proportion" presented in Fig1? The value "0.20" seems the largest level presented, but would that mean, for example, a response "Vastness" occupied approximately 20% of the case when the "Scenery" was described as the eliciting situation?

 Just a check: on Table 3, there seem to be multiple "can(de-ki-ru)"s observed across topics, and in some cases, its Chinese character version is present with the same English counterpart (at Humanity). Is this normal for this technique?

 I was wondering to what extent "the self (as) represented as being non-substantial, denial, and embedded as default in Japan, a country with Buddhist and Shinto backgrounds (p3)" is related to the interdependent views of the self (Markus & Kitayama, 1991). When the self is understood as socially conferred, it may render non-substantial in order to preserve harmony in one's in-group, denied so as to catch up with the group-held norm, and embedded in the group in which one should be a part.

 Typo:

 P5: internal consistent

Is the work clearly and accurately presented and does it cite the current literature?

Yes

If applicable, is the statistical analysis and its interpretation appropriate?

I cannot comment. A qualified statistician is required.

Are all the source data underlying the results available to ensure full reproducibility?

Partly

Is the study design appropriate and is the work technically sound?

Partly

Are the conclusions drawn adequately supported by the results?

Partly

Are sufficient details of methods and analysis provided to allow replication by others?

Partly

Reviewer Expertise:

Cross-cultural psychology

I confirm that I have read this submission and believe that I have an appropriate level of expertise to confirm that it is of an acceptable scientific standard, however I have significant reservations, as outlined above.

## References

[1] : Culture and the self: Implications for cognition, emotion, and motivation. *Psychological Review* .1991;98(2) : 10.1037/0033-295X.98.2.224 224-253 10.1037/0033-295X.98.2.224

# Response

**Published:** 2023-10-10

**Authors:** Ryota Takano

**Affiliations:** Social Psychology, The University of Tokyo, Bunkyo-ku, Tokyo, Japan

**Response to Reviewer #1:**

 I enjoyed reading the manuscript a lot. As it is deals with the cultural generalization of the AWE experience measure, especially in a culture where previous studies showed some interesting cultural differences between the West (Nakayama et al., 2020), the scale standardization should contribute to the test of universality of the emotion.

 My comments are listed below. They are mostly on the logic building up to the interpretation of the main results.

**Reply**: Thank you so much for your valuable comments and queries. We have incorporated all your comments and revised the manuscript. Please find the point-by-point responses and the corresponding revised text below.

**Comment 1.**

 I understand the authors are trying to make the case that the newly translated J-AWE-S has reliability and validity, including factorial and criterion-related.

 However, the current unfolding seems to make an impression that the correlations with DPES are given post-hoc interpretations.

 In other words, how can the validity correlations with DPES (or the lack thereof with self-loss and accommodation) be understood as supporting the validity of the J-AWE-S?

 Similarly, what was the logic behind controlling other positive emotions of DPES to arrive at the regression result of selected positive emotions explaining seemingly different aspects of J-AWE-S as in Table 2?

**Reply**: Thank you for your comment. Since no previous studies have longitudinally examined the relationship between the trait and state aspects of awe, it is unclear how the tendency to feel awe on a daily basis (awe factor of the DPES) is associated with state responses to experiencing actual awe (AWE-S).

 However, several studies have shown that awe-related phenomena observed via the awe factor of the DPES (dispositional awe) could also be seen through the measurement of state-awe (Bai et al., 2017; Piff et al., 2015; Stellar et al., 2018). Hence, we investigated the relationships between the DPES and Japanese AWE-S to verify its construct validity.

 In addition, we controlled other positive emotions of the DPES to examine specific relationships between dispositional awe and each factor of the Japanese AWE-S.

 We have incorporated your comment by adding the following sentences in the last paragraph of the Introduction section.

 (Added sentences) *We used the Dispositional Positive Emotion Scale (DPES) since previous studies demonstrated that awe-related phenomena observed via the DPES could also be seen through the measurement of state-awe ( Bai et al., 2017; Piff et al., 2015; Stellar et al., 2018). We controlled other positive emotions to examine specific relationships between dispositional awe and each factor of the Japanese AWE-S*

**Comment 2.**

 Regarding J-AWE-S, Item 30 seems to show low factor loading. Would removing it contribute to the improvement of the fit?

**Reply: **When item 30 was removed from the factor analysis, goodness-of-fit was slightly improved.

 With item 30: χ ^2^ (390) = 1070.31, CFI = .905, GFI = .854, RMSEA = .062, SRMR = 0.068

 Without item 30: χ ^2^ (362) = 931.34, CFI = .919, GFI = .867, RMSEA = .059, SRMR = 0.053

 Thus, we did not remove it to make international comparisons using the AWE-S easier.

**Comment 3.**

 DPES seems to show a marginal fit. Does it reliably measure the 7 positive emotions? Or, removing some items/emotions increases the overall reliability, and that makes a better test?

**Reply**: The goodness-of-fit of the DPES in this study fitted well as those in previous studies, such as Sugawara et al.’s study (2020).

 This study: χ ^2^ (644) = 1728.55, CFI = .860, RMSEA = .073

 Sugawara et al.'s study (2020): χ ^2^ （644）＝ 4171.89, CFI＝.851, RMSEA＝.081

 With the exception of one item, 38, whose item loading was below .40, the results were significantly unchanged (χ ^2^ (608) = 1650.09, CFI = .864, GFI = .769, RMSEA = .074, SRMR = .063).

 In addition, correlation analysis with each of the awe factors yielded similar results.

- DPES item 4: I feel wonder almost every day (私はほとんど毎日驚嘆の念を感じる)
  - Time: *r *= .18*, Self-loss: *r* = .01, Connectedness: *r* = .30*, Vastness: *r* = -.04, Physiological: *r* = .15*, Accommodation: *r* = .10
- DPES item 16: I often feel awe (私はよく畏敬の念を感じる)
  - Time: *r *= .28*, Self-loss: *r* = .06, Connectedness: *r* = .27*, Vastness: *r* = .09, Physiological: *r* = .14*, Accommodation: *r* = .04
- DPES item 17: I seek out experiences that challenge my understanding of the world (私は世界を自分なりに理解するための経験を探し求めている)
  - Time: *r *= .27*, Self-loss: *r* = .09, Connectedness: *r* = .26*, Vastness: *r* = .14*, Physiological: *r* = .16*, Accommodation: *r* = .11
- DPES item 22: I see beauty all around me (私は自然の美しさを見る機会が多い)
  - Time: *r *= .27*, Self-loss: *r* = .02, Connectedness: *r* = .21*, Vastness: *r* = .09, Physiological: *r* = .05, Accommodation: *r* = -.10
- DPES item 31: I often look for patterns in the objects around me (私はしばしば自分の周りのものの中にパターンを探す)
  - Time: *r *= .19*, Self-loss: *r* = .17*, Connectedness: *r* = .16*, Vastness: *r* = .13*, Physiological: *r* = .13*, Accommodation: *r* = .14*
- DPES item 36: I have many opportunities to see the beauty of nature (私は自分の周りのすべてに美しさを見出す)
  - Time: *r *= .25*, Self-loss: *r* = .02, Connectedness: *r* = .35*, Vastness: *r* = .09, Physiological: *r* = .15*, Accommodation: *r* = .02

Thus, even if there were problems with the factor structure of the DPES, our conclusions would not change.

**Comment 4.**

 As the text analysis of the experience topic and its correspondence with J-AWE-S response is so interesting, if the main aim of the study is to validate the scale in conjunction with the former, authors may present some expected findings given the nature of the J-AWE-S. This seemed possible a-pri-ori, when authors argued that awe involves vastness in scenes or has cultural roots of selfhood.

**Reply**: At first, we did not plan to perform a Structural Topic Modeling (STM). As mentioned in the Method section, the STM were exploratory and not pre-registered. After data were collected, improvement in the first authors’ statistical knowledge and skills enabled us to conduct the STM. Therefore, it was difficult to discuss any predictions of the textual analysis a-priori in the Introduction section.

 However, as pointed out in Comment 5, the findings that “Scenery” and “Threat” were the two most common topics were interesting regarding the cultural characteristics of the awe experience in Japan. It would be important to discuss these results.

 The narrative of the awe experiences included many elements of “Scenery” (landscapes, mountain, sea), which was associated with the vastness factor of the Japanese AWE-S. These results were consistent with the Japanese view of the self (non-substantial, denial, embedded), which was thought to be related to nature worship prevalent among Japanese people (recognition of the self as a part of deified nature).

 In addition, the high proportion of “Threat” ( *ifu*, earthquake, typhoon) was thought to be associated with negative aspects of awe, threat-awe, which was in line with the fact that natural disasters occurred frequently in Japan due to its climate and topography. Since even a positive awe manipulation (recall and describe their experiences of positive awe) evoked the feelings of *ifu*, negative awe in Japanese, sufficiently (Takano & Nomura, 2023), Japanese people could be more likely to feel threatened during awe experiences due to geographic and historical background in Japan.

 Thus, we have incorporated your comment by adding the following sentences in the Discussion section.

 (Added paragraphs) *It should be also noted that* *“* *Scenery” was the most common topic, which was positively related to the vastness factor of the Japanese AWE-S. This topic mainly consisted of nature-related words, such as “landscape,” “mountain,” and “sea.” Previous studies have demonstrated that many Japanese people are familiar with nature worship, a belief that the self is a part of deified nature that is cultivated under the syncretism of Shinto and Buddhism ( Sugimoto, 2020; Takano & Nomura, 2022a). Therefore, these results might reflect the Japanese view of the self in relation to nature worship (i.e., non-substantial, denial, embedded).*

*Interestingly, “Threat,” the second common topic, might be associated with negative aspects of awe (i.e., threat-awe), which is in line with the fact that natural disasters occur frequently in Japan due to its climate and topography. Previous studies suggested that feelings of awe that are triggered by threatening stimuli such as natural disasters could be tinged with the perception of threat ( Takano & Nomura, 2022b). Given that even the recollection of awe experiences based on examples that do not include negative elements (e.g., seeing aurora) evokes the feelings of ifu, negative awe in Japanese ( Takano & Nomura, 2023), Japanese people might be more likely to feel threatened during awe experiences due to Japan’s geographic and historical background.*

**Comment 5.**

 How do we make of the "Expected Topic Proportion" presented in Fig1? The value "0.20" seems the largest level presented, but would that mean, for example, a response "Vastness" occupied approximately 20% of the case when the "Scenery" was described as the eliciting situation?

**Reply**: Thank you for your comment. The expected topic proportion represents what proportion of the total documents (responses) fall into each topic. For example, the value “0.20” indicates that approximately 20% of the responses fell into the topic of “Scenery,” regardless of their association with each of the AWE-S factors.

 We have incorporated this comment by adding statements in *Note* of Figure 1 as below in italic.

 (Added sentences) Expected topic proportions, *proportion of the total documents (responses) falling into each topic*, are depicted by dot size.

 In addition, these results themselves would provide meaningful insight regarding the cultural characteristics of awe experiences in Japan. Therefore, we modified the Results section by adding some results as shown below in italic, which were also discussed in the Discussion section (for details, please see the Reply to Comment 4).

 (Added sentences) Furthermore, seven topics were generated by the STM and labeled: “Spirituality,” “Threat,” “Spatiality,” “Universe,” “Scenery,” “Humanity,” and “Aesthetics” (see Figure 1). The top 20 highest probability words are listed in Table 3. *In particular, “Scenery” and “Threat” were the two most common topics that emerged in all the responses. Regarding the Japanese AWE-S,* participants with higher scores on the time factor were more likely to use words related to “Spatiality” and “Scenery.”

**Comment 6.**

 Just a check: on Table 3, there seem to be multiple "can(de-ki-ru)"s observed across topics, and in some cases, its Chinese character version is present with the same English counterpart (at Humanity). Is this normal for this technique?

**Reply**: The word “de-ki-ru” written in the Chinese character was owing to conversion errors when Table 4 was created. We have corrected all the mistakes to “de-ki-ru” in Hiragana (please see Table 4).

**Comment 7.**

 I was wondering to what extent "the self (as) represented as being non-substantial, denial, and embedded as default in Japan, a country with Buddhist and Shinto backgrounds (p3)" is related to the interdependent views of the self (Markus & Kitayama, 1991). When the self is understood as socially conferred, it may render non-substantial in order to preserve harmony in one's in-group, denied so as to catch up with the group-held norm, and embedded in the group in which one should be a part.

**Reply**: Nomura et al. (2020) stated that these characteristics of the sense of self based on Japanese religious and historical backgrounds were consistent with Markus and Kitayama's (1991) theory in cultural psychology. To attract a wider range of readers, we have added the following sentences in the Introduction section.

 (Added sentences) *Similarly, Markus and Kitayama (1991) advocated independent and interdependent self-construals. Independent self-construals, more widespread in North American and Western European cultures, prioritize an individual’s needs, whereas interdependent self-construals, more common in Asian, Latin American, and African cultures, prioritize one’s social interactions.*

**Comment 8.**

 Typo P5: internal consistent

**Reply**: Thank you for your feedback. We have modified the words accordingly.
